# Solar ultraviolet light collector for germicidal irradiation on the moon

**DOI:** 10.1038/s41598-023-35438-4

**Published:** 2023-05-23

**Authors:** Matteo Lombini, Laura Schreiber, Roberto Albertini, Elisa Maria Alessi, Primo Attinà, Andrea Bianco, Enrico Cascone, Maria Eugenia Colucci, Fausto Cortecchia, Vincenzo De Caprio, Emiliano Diolaiti, Mauro Fiorini, Luigi Lessio, Alberto Macchi, Giuseppe Malaguti, Giuseppe Mongelluzzo, Giovanni Pareschi, Maria G. Pelizzo, Cesira Pasquarella

**Affiliations:** 1 Istituto Nazionale di Astrofisica - Osservatorio di Astrofisica e Scienza dello Spazio di Bologna, Bologna, Italy; 2 Dipartimento di Medicina e Chirurgia, Università di Parma, Parma, Italy; 3 Istituto di Matematica Applicata e Tecnologie Informatiche “E. Magenes” - Consiglio Nazionale delle Ricerche, Milan, Italy; 4 Istituto Nazionale di Astrofisica - Osservatorio Astronomico di Brera, Merate, LC Italy; 5 Istituto Nazionale di Astrofisica - Osservatorio Astronomico di Capodimonte, Naples, Italy; 6 Istituto Nazionale di Astrofisica - Istituto di Astrofisica Spaziale e Fisica Cosmica di Milano, Milan, Italy; 7 Istituto Nazionale di Astrofisica - Osservatorio Astronomico di Padova, Padua, Italy; 8 Dipartimento di Ingegneria dell’Informazione, Università di Padova, Padua, Italy

**Keywords:** Infectious diseases, Solar energy and photovoltaic technology, Pathogens

## Abstract

Prolonged human-crewed missions on the Moon are foreseen as a gateway for Mars and asteroid colonisation in the next decades. Health risks related to long-time permanence in space have been partially investigated. Hazards due to airborne biological contaminants represent a relevant problem in space missions. A possible way to perform pathogens’ inactivation is by employing the shortest wavelength range of Solar ultraviolet radiation, the so-called germicidal range. On Earth, it is totally absorbed by the atmosphere and does not reach the surface. In space, such Ultraviolet solar component is present and effective germicidal irradiation for airborne pathogens’ inactivation can be achieved inside habitable outposts through a combination of highly reflective internal coating and optimised geometry of the air ducts. The Solar Ultraviolet Light Collector for Germicidal Irradiation on the Moon is a project whose aim is to collect Ultraviolet solar radiation and use it as a source to disinfect the re-circulating air of the human outposts. The most favourable positions where to place these collectors are over the peaks at the Moon’s poles, which have the peculiarity of being exposed to solar radiation most of the time. On August 2022, NASA communicated to have identified 13 candidate landing regions near the lunar South Pole for Artemis missions. Another advantage of the Moon is its low inclination to the ecliptic, which maintains the Sun’s apparent altitude inside a reduced angular range. For this reason, Ultraviolet solar radiation can be collected through a simplified Sun’s tracking collector or even a static collector and used to disinfect the recycled air. Fluid-dynamic and optical simulations have been performed to support the proposed idea. The expected inactivation rates for some airborne pathogens, either common or found on the International Space Station, are reported and compared with the proposed device efficiency. The results show that it is possible to use Ultraviolet solar radiation directly for air disinfection inside the lunar outposts and deliver a healthy living environment to the astronauts.

## Introduction

The space exploration programs for the near future involve bringing humans back to the Moon’s surface. In particular, the Artemis program by NASA aims to get the first woman and the next man on the Moon by 2024 for the first long-term mission^1^. An established target for different agencies and organizations is to colonise the Moon and build outposts on the lunar surface^2^. In the longer run, the goal is to carry humans to Mars: the experiments that will be carried out on the Moon are, in part, to support future Mars missions. The long duration and exploration of human spaceflight pose many significant challenges exposing astronauts to environments with uncertain and unknown risks to their health. Biological, chemical and physical potential hazards are posed at each phase of a mission^3–6^. Currently, the International Space Station (ISS), staffed continuously since the first resident crew entered the facility on 2 November 2000, is the only orbital living and working environment outside the Earth’s atmosphere. Studies carried out inside the ISS refer to potential health risks during spaceflights^7–9^. Publications and reports from experiments aboard the Chinese Tiangong space station, crewed since 2021, are expected in the next years^10^. Publications from other shorter-term spacecraft, such as the Space Shuttle, are available^8,11^ Among health considerations, risks are posed by exposure to airborne environmental, biological and chemical contaminants onboard spacecraft, which could be the same inside the future Moon’s habitable modules. Biological contaminants can be related to infections, allergies, and toxic effects. Despite most microorganisms do not threaten human health and will likely play an essential role (e.g., waste remediation, water and air purification, food sources on long-term missions), microorganisms may produce adverse effects on the health of crew members, due in particular to the immune system deficiency of astronauts^12^ and changes of molecular and biochemical characteristics of microorganisms^13–15^.

To reduce the possibility of indoor contamination onboard spacecraft, preventive measures are currently performed: health checks of astronauts before departure, vaccinations, quarantine, microbiological control of food, control of the material sent on board, personal hygiene improvement activities, environmental disinfection^7,11^. Different devices coupled or not with Heating, Ventilation, and Air Conditioning (HVAC) systems can be used for air disinfection. Among the numerous disinfection methods that have been developed, we will focus on Ultraviolet Germicidal Irradiation (UVGI) through UVC light (200–280 nm), which inactivates many microorganisms, such as viruses, bacteria, protozoa, fungi, yeast, and algae^16,17^. Upon UVC absorption, the pyrimidines in RNA or DNA are converted mainly into pyrimidine dimers (but it also breaks the crosslink between nucleic acids and proteins). If the population of dimers is sufficiently high, transcription errors occur, ultimately resulting in the inactivation of microorganism replication. UVC irradiation’s efficiency in inactivating microorganisms depends on several factors since the required dose depends on the factors intrinsic to different microorganisms to UVC light^18–20^. Moreover, the inactivation rate depends on the irradiation wavelength^21^, distance from the source^22^, exposure time^23^, Relative Humidity (RH)^24,25^, and an adequate filtration of dust^26^, which absorbs and scatters light, shielding pathogens. In this framework, authors have been carrying out studies on highly effective UVGI devices which exploit the concept of power density enhancement of the UVC sources inside a volume (the air duct) due to a high reflectivity of the internal surfaces^27–31^. Differently from water, air components are very transparent at the employed wavelengths^32^ and the UVC absorption by the pathogens is minimal thanks to their very low concentration^33^. Since no secondary effects are produced, the UVC light dose can be administered ’in pieces’ after any of the numerous internal reflections, according to the Bunsen and Roscoe law^34^. Differently from Earth-based applications, where the UVC sources are artificial (e.g., Mercury vapour lamps or LEDs), our idea is to use, for the first time, the UVC component of the solar radiation directly as a source^35^ for air disinfection inside the habitable module of the lunar outposts^36^. The Solar ultrAvIolet Light cOllector for GeRmicidal irradiation on the Moon (SAILOR Moon) is a project where the UVC component of the Sun’s radiation is collected and becomes the source for UVGI, made possible due to the peculiarity of the Moon’s poles relative to sunlight extended exposure. By exploiting the enhanced power inside the air duct produced by highly reflective internal surfaces, it is possible to obtain enough power to inactivate effectively airborne pathogens. The study’s objectives were to demonstrate, although through simulations, that it is possible to obtain an effective pathogens’ inactivation by using the UVC band of the solar radiation uniquely. Moreover, we have introduced a new concept of static solar concentrator for a specific application at the Moon’s poles, which appears to be very effective. We hope to bring a possible alternative to the current or proposed disinfection systems for lunar habitable modules and, more generally, for prolonged human missions in outer space. The present paper is organised as follows: a description of potential hazards for long-term permanence in space and a short review of the pathogens found aboard the ISS is given. Then, the solar irradiance conditions on the Moon and the SAILOR Moon concept are described, and the performance of the pathogen’s inactivation efficiency through optical and CFD simulations are reported. The simulation results are compared with the required UV dose for some airborne pathogens. While this paper’s goal is to present a novel idea to the scientific community, the study was done assuming some model simplifications, described at the end of the “Results and discussions” Section.

### Environmental conditions and target pathogens

Potential health risks during spaceflights include short-term health consequences from being in microgravity (e.g., nausea, blurred vision), as well as long-term health consequences that arise or continue months or years after a flight (e.g., radiation-induced cancers, loss of bone mass)^6,12^. Astronauts are in a long time under microgravity conditions and are exposed to immune system compromise. Microgravity determines the alteration of the distribution of circulating leukocytes, the production of cytokines, the function of Natural Killer and T cells, granulocyte function, levels of immunoglobulins, virus-specific immunity and an increased reactivation of latent viruses^14,37–42^. Moreover, astronauts are exposed to alteration of the commensal microbial population, reduction of anaerobic microorganism’s presence and increase of aerobic Gram-negative bacteria and staphylococci on the skin, upper respiratory tract, and colon^43–48^. Furthermore, there are environmental alterations that modify the replication and virulence of microorganisms, such as increased exponential growth, higher minimum inhibitory concentrations towards the various classes of antimicrobial agents, increased biofilm formation, and increased survival within macrophages^14,15,49–53^. In these conditions, all microorganisms should be considered as potentially pathogenic to humans. Microorganisms can also determine the damage to materials; studies performed on Mir and ISS indicated that some equipment and structural materials were prone to the accumulation and proliferation of bio-destructive bacteria and fungi^54,55^. Damage to polymers and metals could be observed. This resulted in malfunctioning, and even breakage of specific units, e.g., air conditioners, water recycling systems, etc., and degradation of the spacecraft’s critical materials, which may result in system failure and endangering crews^7^. Infections of crew members or health issues related to the pathogenic action of microorganisms have been reported only rarely^56^. Crew members are the primary source of microorganisms, capable of eliminating many particles (potentially carrying biological agents) in the environment both through the desquamation of the skin and the acts of coughing, sneezing, speaking, breathing, etc., in an environment made more complex by microgravity^42,44,57–62^ and the impossibility to exchange with primary air. Data obtained from the Apollo^39^, Skylab^8^, space shuttle^50^, and the Russian space station Mir^40,49^ confirm that space environments are compatible with human occupation. However, biological payloads, resupply vehicles, hardware and supplies, and food or plant material are additional sources of microorganisms^63^.

Microorganisms are ubiquitous throughout the habitable modules of spacecraft^47,50^, and, in closed environments in microgravity conditions, they will spread everywhere for a long time^7,9,54,64^.

The environmental biocontamination of the ISS has been followed up on since its early construction days and has been under surveillance since its first inhabitation. The main emphasis has been placed on the air quality and the surface contamination of internal structures^8,65,66^. Monitoring the microbial community onboard the ISS is essential to assess risk factors for crew members’ health and evaluate the material integrity of the spacecraft^8,65,66^. Since the beginning of the ISS, routine microbial monitoring of surfaces, air, and water has occurred using culture-based techniques^11,67^. However, only a tiny fraction of organisms can be detected using culture-based analysis, limiting the understanding of the diversity of microbes^67^. Therefore, molecular methods are being developed for their use on ISS, such as quantitative polymerase chain reaction (qPCR) and targeted amplicon sequencing, which can identify and quantify both culturable and unculturable organisms and provide a more thorough assessment of what is present and in what amounts^61,68–71^. Anyway, microbial monitoring of the ISS with molecular-based methods is not routinely used because of the lack of simple, compact, and reliable sample processing instruments onboard the ISS^65–68^. Moreover, new approaches (i.e., New Generation Sequencing-NGS, proteomic, real-time PCR) have been applied, dealing with real-time monitoring^61,68–71^. In this contest, we can take advantage of the knowledge in health care facilities, operating theatres, pharmaceutical, food and electronics industry, and cultural heritage, supported by previous experiences in spacecraft, MIR, and ISS missions^11,65,66,72–75^ and, also, in periodically confined Antarctic base Concordia, were prolonged confinement of the crew resulted in increased airborne contamination associated to human activity^76,77^. The ISS microbiome was not found to be stable in composition and diversity, although a core microbiome persists over time independent of the individual crew microbiome. All core microbiome genera have also been found in ISS dust samples from 2004 and 2008, as well as other ISS microbiome studies, indicating that this core microbiome is indeed established onboard the ISS^56^. Moreover, a genomics-based meta-analysis demonstrated that although pangenomes of *Bacillus* and *Staphylococcus* isolated from the ISS differed from Earth-based counterparts, these differences did not appear to be health threatening^78^. Bacterial species found in the ISS are most associated with the oral microbiome, and human upper respiratory tract^61^. The primary source of airborne fungi may be food or plant material. The main bacterial phyla detected onboard the ISS in air and on surfaces, by either cultivation or molecular methods, were* Staphylococcus (Firmicutes)*, *Corynebacterium*, and *Propionibacterium (Actinobacteria)*^68^. In cultivation-based assays,* Bacillus* and *Staphylococcus* species were the most detected *Firmicutes*, whereas *Staphylococcus *utterly dominated the *Firmicutes*-affiliated signatures detected by molecular methods. The most probable reason for this observed discrepancy might be the disability of standard DNA isolation protocols to open spores adequately^79^. Bacteria belonging to the *Staphylococcus* sp. *genus* were isolated from 84% of the surface samples; the two seconds most identified genera were *Bacillus* sp. (31.7%) and *Corynebacterium* sp. (9.4%)^65^. The prevailing species found on surfaces were *Staphylococcus auricularis*, *S. epidermidis *(22.4%)^9^. *Bacillus sphaericus* and *S. hominis*, encountered in 23.4%, 22.4%. 12.1 and 9.3% of the cases, respectively. Species with opportunistic pathogenic behaviour were isolated as well (*B. cereus*, *Eikenella corrodens*, and *S. aureus*)^9^. Moreover, *Flavobacterium indologenes*, *Pseudomonas putida*, and* Xanthomonas malthophila*, that can cause materials biodeterioration, were detected^43,44^. Concerning fungi, a higher abundance of *Aspergillus* and *Penicillium* onboard the ISS were detected either by cultivation or by using other detection approaches^65,79,80^. Inside the ISS Japanese Kibo module, after a year of operations, no *Penicillium* but skin-associated *Malassezia* was detected^81^. *Aspergillus* sp., *Penicillium* sp., and *Saccharomyces* sp. were the most common genera. Some samples contained *A. versicolor* and* Cladosporium* sp. are known for their capacity to colonise natural and synthetic polymers. Inevitably, the ISS will also be home to an unknown number of microorganisms^65^. Regarding viral contamination, a recent review^42^ reports 72 different virus genera identified, from 21 families, including the ones that contain human pathogens. It is also worth noting that the metagenomic analysis was performed only on the pooled subset of environmental samples with a 126bp average length of reads; therefore, some viruses might have been missed during the study^42^. Moreover, the viral genomes are underrepresented in genomic databases that assign sequences, so a significant portion may remain unidentified^42^. Reads similar to animal viruses were distributed into 33 genera, 13 known to infect humans and cause diseases of varying severity, including a range of herpesviruses, which establish latency and can undergo reactivation^8^. Pathogenic viruses were present in low abundance and unlikely to cause significant health problems on short-term space missions, even under conditions unfavourable to a healthy immune system. However, their impact on long-term missions remains unknown^8,82^. Table 3 shows some airborne microorganisms. The required inactivation doses, reported from literature for all considered microorganisms, have been considered references when dimensioning the system and calculating its sanitising performance through simulations.

### Sun’s UV irradiance and ephemeris at the lunar poles

**Figure 1:**
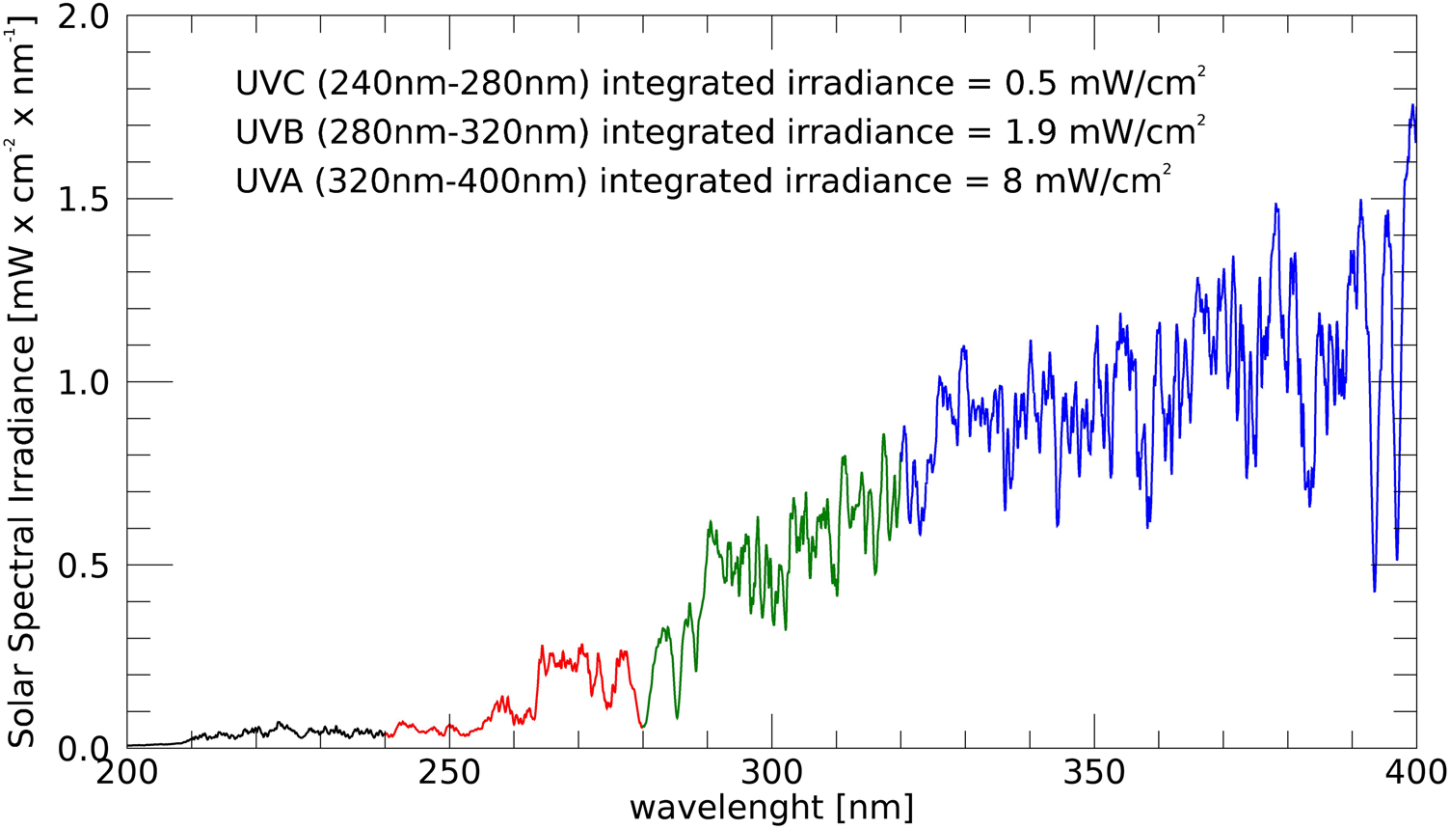
Spectral solar irradiance in the Ultraviolet band from the SOLar SPECtrometer on board the ISS. The red part of the curve is the reduced UVC bandwidth used for the SAILOR Moon efficiency simulations since ozone formation inside the air duct would occur for light with $\lambda<$ 240 nm.

The solar irradiance outside of the Earth’s atmosphere has been measured in the framework of the SOLar SPECtrometer (SOLSPEC) instrument^83^ of the SOLAR payload on board the ISS. Figure 1 shows the spectral irradiance extracted from the SOLSPEC data archive in the whole UV band (200–400 nm). According to Biasin et al.^84^, and Beck et al.^85^, the spectral region between 240 and 280 nm can be considered to have the same germicidal efficiency. At lower wavelength, it is supposed to be the same^86,87^, but it has not been considered in SAILOR Moon efficiency simulations due to ozone formation inside the air ducts, which is toxic^88^. We have considered only the 240–280 nm bandwidth, with an integrated irradiance of 0.5 $\textrm{mW}/\mathrm{cm^2}$, for the efficiency calculations of the UVGI. In the UVB (280– 320 nm) and UVA (320–400 nm) bands, the disinfection efficiency drops but the solar irradiance increases. Therefore, we could expect an effect that is worth exploiting. Moreover, studies suggest that a synergic combination of UVC and longer wavelength could increase the inactivation rate^89,90^, but this is left for future investigation.

The choice of the Moon for the proposed device comes from its low obliquity with regard to the ecliptic plane, about 1.5$^\circ$. This means that at polar latitudes, the maximum elevation that can be achieved by the Sun is 1.5$^\circ$. Hence, the poles are thought to harbour, inside the craters, permanently shadowed regions where water ice might have been trapped (see, e.g. Hayne et al.^91^ and references herein). The percentage of sunlight received by a given area is, as a matter of fact, dependent on the tilt of the spin axis and the topography of the region^92^. Because of the need and interest in establishing a lunar base close to where water ice can be found, extensive studies have been devoted to understanding if topographic features are associated with a long enough solar illumination in the same regions^93^ to ensure robotic and human-crewed operations. The data obtained especially by Kaguya^94^ and LRO^95–97^ missions confirmed the existence of the so-called “peaks of eternal light”, called in this way in 1880 by Flammarion^92^: [French/English] Aux pôles lunaires (où l’on ne voit d’ailleurs ni neiges ni glaces), il y a des montagnes si étrangement situées, que leur cime ne connaît pas la nuit; *jamais* le Soleil ne s’est couché pour elles! On peut les appeler *les montagnes de l’éternelle lumière* / At the lunar poles (where indeed we cannot see snow or ice), there are mountains so strangely situated that their peak does not know the night; the Sun has *never* set for them! We can call them *the peaks of eternal light*”. They are rims of given craters and ridges lit for a large portion of the year. The accurate estimates are obtained by considering the low obliquity (and thus the negligible seasonal variations), the topography of the features at the poles and the axial lunar precession (the spin axis rotates in about 18.6 years). According to Gläser et al.^97^, the best candidates in terms of average illumination percentage over 20 years at the North Pole are the equator-facing rims of Hinshelwood, Peary and Whipple craters, while at the South Pole the Shackleton crater and two regions on Connecting Ridge. The corresponding percentage ranges from about 70% up to 83%. The maximum time in shadow varies instead from nearly 100 hours to 335 hours. These values are less optimistic according to Speyerer and Robinson^98^, who, however, analysed one year. NASA^36^ has selected the following 13 sites at the South Pole as candidates for an Artemis III lunar landing: Faustini Rim A, Peak Near Shackleton, Connecting Ridge, Connecting Ridge Extension, de Gerlache Rim 1, de Gerlache Rim 2, de Gerlache-Kocher Massif, Haworth, Malapert Massif, Leibnitz Beta Plateau, Nobile Rim 1, Nobile Rim 2 and Amundsen Rim. The choice was driven by the fact that they can ensure continuous access to sunlight throughout 6.5 days.

## Materials and methods

### SAILOR Moon design

The SAILOR Moon project is a study on a service module for re-circulated air disinfection, through solar UVC radiation, inside the future lunar habitable modules. As explained in the previous section, the lunar poles are the most favourable locations in outer space due to the unique prolonged solar irradiation and the limited range of the Sun’s apparent position around the horizon. We present two possible solar UVC light concentrators, which produce the germicidal source for the air inside the habitable modules. We have considered two sunlight collectors: (i) a Sun’s tracker, which can be mounted on a classical tracking mechanism; (ii) a static collector. Accordingly to the previous section, the slight Moon’s axis tilt and the positioning of possible landing sites in proximity of the poles make the Sun’s apparent position confined to ± 2$^\circ$ around the horizon, along the Zenith angle. Considering the Sun’s angular size ($\approx$ 30 arcmin) and some contingency, we have considered the sunlight collectors described in the following to be able to collect light in a ± 3.5$^\circ$ range around the horizon, to be conservative (i.e. pointing accuracy).

At the moment of writing, no requirements for air flux or air duct size exist for lunar habitats. The only available data refer to the ISS^99^, regarding the air flux of the re-circulating air (460 $\mathrm{m^3}/\textrm{h}$) and the air duct diameter (14 cm). These two quantities have been used as simulation parameters. The other parameters, listed in Table 1, have been chosen arbitrarily but are considered reasonable. Anyway, the efficiency results, shown in the “Results and discussions” Section, can either be scaled linearly (with the collecting area, for example), or some indications on the efficiency trend with parameter variations will be given.

#### Sun tracking concentrators

**Figure 2:**
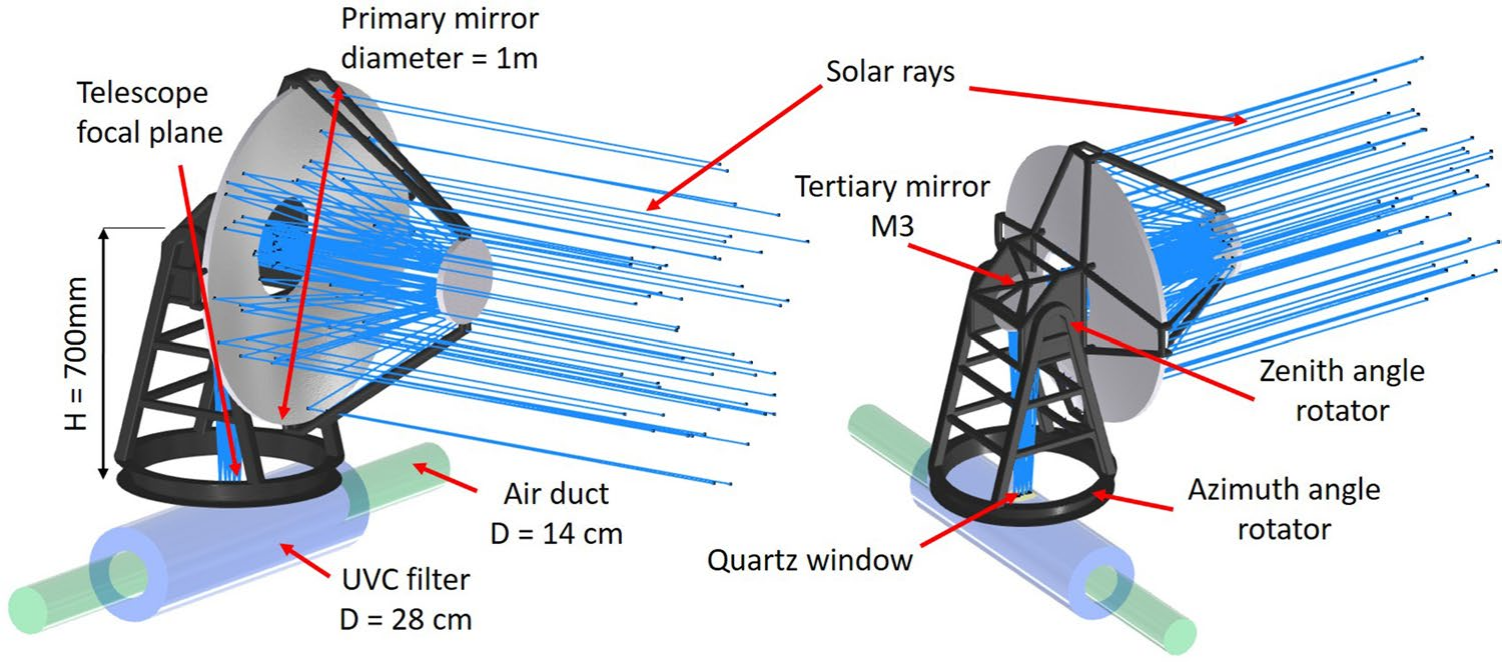
Proposed design for a possible Sun’s tracking concentrator: Ritchey-Chretien type telescope. A tertiary flat mirror behind the telescope aperture compensates for Zenith angle variations and maintains the focal plane fixed over the quartz window of the air duct.

The simplest optical solution for an efficient light concentrator of a moving source is a small Field of View (FoV) concentrator with a tracking mechanism. The design presented in Fig. 2 represents a possible example of optical configuration without claiming to be the most efficient solution. Other designs would be considered in the case of investigation for the actual implementation of the device.

The presented concentrator is a two mirrors Ritchey-Chretien type telescope with a 1$^\circ$ FoV (the Sun’s apparent diameter is about 0.5$^\circ$). The telescope mounting has similarities with the radio telescope mountings since the main goal is the light concentration, not the optical quality on the focal plane. The only requirement would be that the Sun image’s position, size and shape should pass through a quartz window, be transparent to the whole UV range^100^, and become the source for air disinfection inside the air duct. Two motorised rotators track the Sun’s apparent movement. The Zenith angle rotation range is supposed to be ± 3.5$^\circ$ around the horizon, permitting a flat rotating tertiary mirror (M3) to compensate for the focal plane shift due to the declination angle variation to deliver the Sun’s image in the same position above the quartz window. The M3 compensation mechanism could be a simple pantograph leverage system. The variation rate of both Azimuth and Zenith angles is slow enough not to be considered an issue for the Sun’s tracking (less than 0.5 $\mathrm{deg/h}$). A simple Sun sensor would be sufficient to maintain the source inside the telescope FoV, and a stepped tracking mechanism would simplify the system regarding duty cycle control. Even more accurate pointing devices would not be an issue if the concentrator’s goal were to deliver a higher optical quality focal plane. A more stable Sun’s image would permit the coupling of an optical fibre bundle and transport UV light to further distances from the concentrator, in case the air ducts were far from the concentrator or for different applications (water or surface disinfection). This option is beyond the paper’s goal and has not been investigated in detail. The telescope mirrors are considered to have a high reflectivity R. A possible material could be Alanod MIRO UV C^101^, having $R>0.9$ over the UVC range and at longer UV wavelengths, with a smooth surface to avoid scattered light. Another more expensive solution is the deposition of a multilayer coating optimised for UVC.

#### Static concentrator

The peculiarity of the lunar poles concerning the Sun’s apparent position makes possible the use of a static concentrator, able to collect the solar radiation for the whole period of exposure, thanks to the reduced Zenith angular displacement of the Sun’s position.

**Figure 3:**
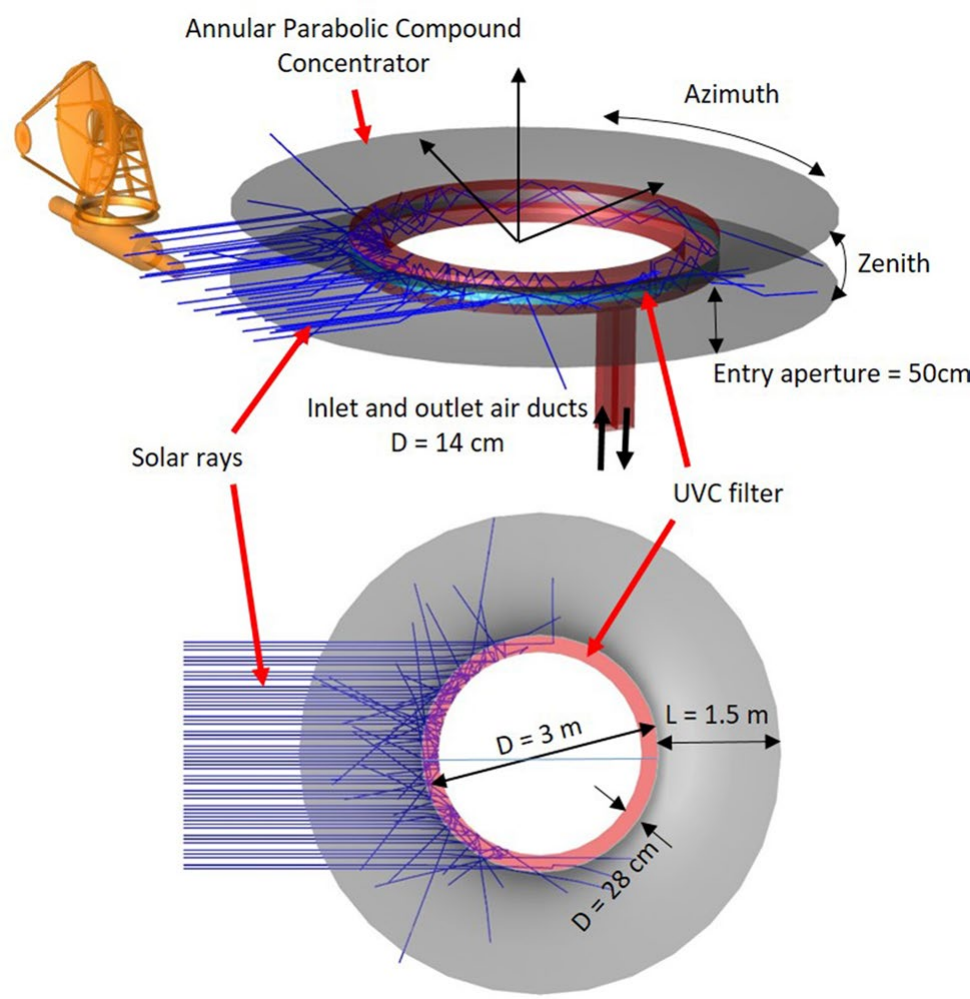
Sketched designs of the Annular Compound Parabolic Concentrator for solar UVC light concentration: side and top views. The image of Sun’s tracking concentrator at the top-left has the purpose to visually show the two systems’ scale. The two configurations sizes has been chosen to deliver a similar overall Fluence, as shown in Table 2.

**Figure 4:**
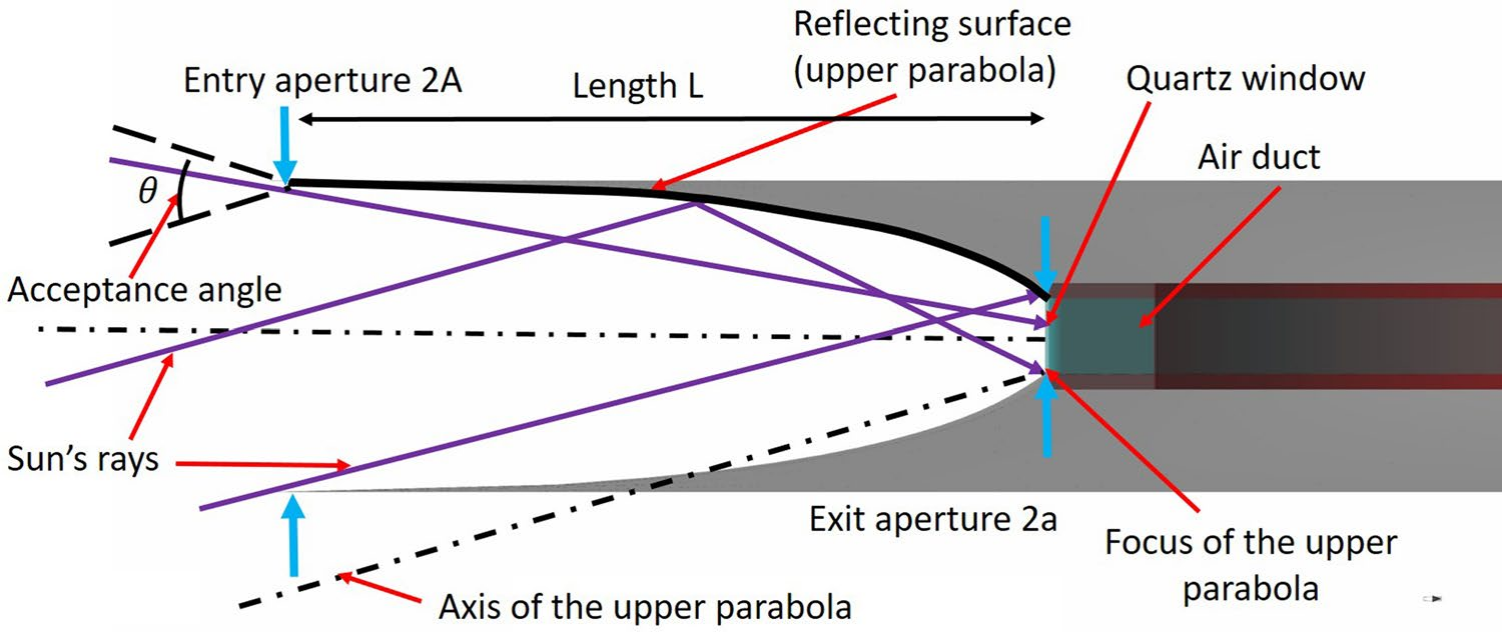
The external profile of the annular Compound Parabolic Concentrator. The parameters refer to the upper side. The lower side would have the same parameter values in case of a symmetrical accepting angle between the two sides. Parameter values are listed in Table 1.

The two images in Fig. 3 show the conceptual design of an annular concentrator. Sun’s light enters inside the red-coloured air duct through the quartz window, which also has an annular shape. The inlet and outlet sections of the air duct are directed downward since the concentrator is supposed to be placed on top of the habitable modules to avoid shadowing. The light inside the air duct undergoes multiple reflections until it is absorbed by the internal surfaces or exits the duct through the window. Ray-tracing simulations performed using Zemax OpticStudio^®^ (see “Optical simulations” Section below) show that more than half of the internal duct volume is filled with solar UVC light. The static concentrator has the external profile of a Compound Parabolic concentrator (CPC)^102^, a non-imaging type light concentrator widely used for water heating and power generation. All the light rays entering the CPC entry aperture with an angle smaller than the acceptance angle $\theta$ are reflected by the parabolic surfaces inside the exit aperture area (Fig. 4). The device’s dimensions, listed in Table 1, can be easily derived by using the “edge-ray principle” applied to the CPC design, described in Tian et al.^103^. At the annular CPC exit aperture, a quartz cylindrical shell acts as an entrance window for the solar UVC radiation to the annular UVC filter. If the outpost location were precisely at the lunar pole, the annular concentrator would have its symmetry axis horizontal. The two parabolas could have axes with different acceptance angles to intercept all Sun’s rays depending on the exact outpost location.

#### Air ducts

SAILOR Moon aims to maximise the germicidal efficiency of the solar UVC radiation inside the air ducts. The quantity to maximise is the *Fluence* (*F*), also called UV dose, which is defined as the total radiant energy from all directions passing through an infinitesimally small sphere of cross-sectional area $\delta$A, divided by $\delta$A, with typical units of $\textrm{mJ}/\mathrm{cm^2}$. Fluence is equal to the Irradiance or Fluence Rate (*FR*), with standard units of $\textrm{mW}/\mathrm{cm^2}$, multiplied by the pathogens’ residence time *t* inside a unit volume. The UVC filter concept relies on the *FR* magnification inside a section of the air duct internal volume due to the multiple reflections of the light rays, thanks to the implementation of highly reflective materials to coat the internal duct surfaces. Possible materials could be Alanod with a coarse substrate^101^, which has *R* > 0.9, or the Polytetrafluoroethylene (PTFE)^104^, which is reported to have an *R* = 0.95 at 275 nm and a Lambertian scattering distribution (all incident rays are diffused with equal probability anywhere in the unit semicircle independently of the incidence angle). As described in Lombini et al.^105^, a Lambertian scattering of the internal surfaces produces the *FR* distribution inside the volume to be smoothed and more uniform. Another strategy to increase the germicidal efficiency of the duct is to act on the pathogens’ residence time. This is possible by optimising the duct geometry. For both the proposed concentrator types, the irradiation zone has a section doubled compared to the inlet and outlet duct section diameter, reducing the airspeed in the filter and consequently increasing the air residence time *t*^106^. The other sides of the air duct are supposed to have the internal sides coated with poorly reflective UV material, even though a more prolonged, highly reflective section would increase the inactivation efficiency. Direct exposure to the UVC light from the duct apertures should be avoided due to its harmful effects on humans^107,108^. For this reason, we have considered a limited duct portion coated with reflective material, which reduces the possibility of light leaks. An optimised UVC filter length will be taken into account for specific application cases. The “Results and discussions” Section briefly discusses the system performance when varying some CPC parameters.

### Pathogens’ inactivation efficiency computation

We have estimated the expected UVC dose delivered to pathogens circulating inside the ducts, by combining computational Fluid Dynamic (CFD) simulations of the particles’ trajectories and velocities, to estimate the local residence time *t*, and the expected volumetric *FR* inside the UVC filter produced by the solar radiation. The inactivation of the pathogens is a function of the total UV energy absorbed. A simplified model^17^ is the exponential relationship:

1 $$\begin{aligned} S = e^{-kF} = e^{-ktFR} \end{aligned}$$

where *e* is the Napier’s constant, *S* is the survival fraction of microorganisms after being exposed to UVC light and *k* is the specific rate constant unique to each type of microorganism ($\mathrm{cm^2}/\textrm{mJ}$). The following sections describe more in detail the performed simulations and the considered parameters.

#### Parameters

Table 1 lists the main parameters used for the simulations. The considered solar UVC irradiance refers to the bandwidth between 240 and 280 nm, while the air flux inside the recirculating ducts is supposed to be 230 $\mathrm{m^3}/\textrm{h}$ or 460 $\mathrm{m^3}/\textrm{h}$ (the last one is the ISS reference value). Concerning the Sun’s tracking concentrator, we have considered $R = 0.9$ for each of the three telescope mirrors and obscuration of 30% of the 1 m diameter primary mirror due to the secondary mirror. The solar UVC light is, therefore, a 2 W source. The F/6 telescope produces a 5 cm size Sun’s image. The irradiation sections have a doubled diameter to the inlet and outlet duct diameter (28 cm vs. 14 cm—see “Air ducts” Section description above). The Sun’s tracking concentrator design has been coupled with a UVC filter having a cylindrical shape of 1 m in length. The filter’s internal reflectivity has been simulated as being $R = 0, 0.9, 0.95, 0.99$, while the other air duct sections are supposed to be coated with a UVC-absorbing material. The Static concentrator has a diameter of 3 m, and the collected power, i.e. the light entering the air duct through the quartz window, is 4.5 W. The annular UVC filter has a squared section 28 cm wide and the same three internal reflectivity values as for the other configuration. These parameters have been refined during simulations to have a reasonable disinfection performance and be used as a starting point for future implementations.

**Table 1:** Main parameters used to perform the CFD and optical simulations.

	Parameter	Value	Unit
	Solar UVC (240–280 nm) irradiance	0.5	
	Air flux	230, 460	$\mathrm{m^3}/\textrm{h}$
Sun’s tracking concentrator	Diameter	100	cm
	Obscuration	0.3	
	Optics throughput	0.7	
	UVC source power	2	W
	diameter	14	cm
	UV filter diameter	28	cm
	UV filter length	200	cm
	Internal reflectivity R	0, 0.9, 0.95, 0.99	
Static concentrator	Accepting angle $\theta$	7	deg
	Annulus diameter	300	cm
	Entry aperture 2A	50	cm
	Exit aperture 2a	10	cm
	Length L	150	cm
	Optics throughput	0.9	
	UV source power	4.5	W
	UV filter side	28	cm
	UV filter length	470	cm
	Internal reflectivity R	0, 0.9, 0.95, 0.99	

#### Fluid-dynamic simulations

Since pathogens are expected to be carried by droplets released by astronauts while breathing/coughing, CFD simulations have been conceived to describe the droplets’ motion inside the air ducts of both SAILOR Moon configurations. The goal was to predict the exposure of droplets to the UV radiation, to be combined with the expected Fluence rate from the optical simulations, and hence the Fluence.

Simulations have been performed using the commercial software Ansys Fluent^®^ (v18.1), considering reasonable conditions for a habitable human environment, i.e. air as a gas, an ambient pressure of 1 atm, an ambient temperature of 25 $^\circ$ $C$. Gravity has been set to the Lunar value (1.62 $\textrm{m}/\mathrm{s^2}$); however, we have performed simulations in different gravity conditions (Earth, Moon, no gravity), which did not alter the droplet dynamics. The initial conditions of the runs have been set in terms of volume flow rate, following the values reported in Table 1. A velocity inlet boundary condition has been set to the inlet section of the duct, with the proper wind speed, to reproduce the requested flow rate. Simulations have considered turbulent flow, as the operating conditions lead to a Reynolds number (Re) greater than 40000 for all simulated cases (turbulence onset is conventionally in the 2000–5000 Re range). For this reason, the realisable $k-\epsilon$ model has been used. Droplets have been simulated as discrete phases and tracked through the particle tracking tools provided by Fluent. They have been considered spherical, made of liquid water, and in size range of 0.5–25 ${\upmu }\textrm{m}$ (in diameter), following the expected size range of bioaerosol^109,110^. In this range, particles may behave differently depending on their size and speed. The different behaviour can be predicted through the Stokes number (*Stk*), a dimensionless number characterising the behaviour of suspended particles in a fluid flow dependent on several parameters, including droplet speed and diameter. For most simulated cases, $Stk < 1$ indicates that droplets tend to follow the fluid streamlines; however, larger grains in the considered interval have $Stk > 1$ in some simulations, showing the tendency to separate from the primary fluid flow. Due to the assumed slow rates, the different size particles showed very little difference in the velocity and behaviour inside the duct, making the result independent of their size.

**Figure 5:**
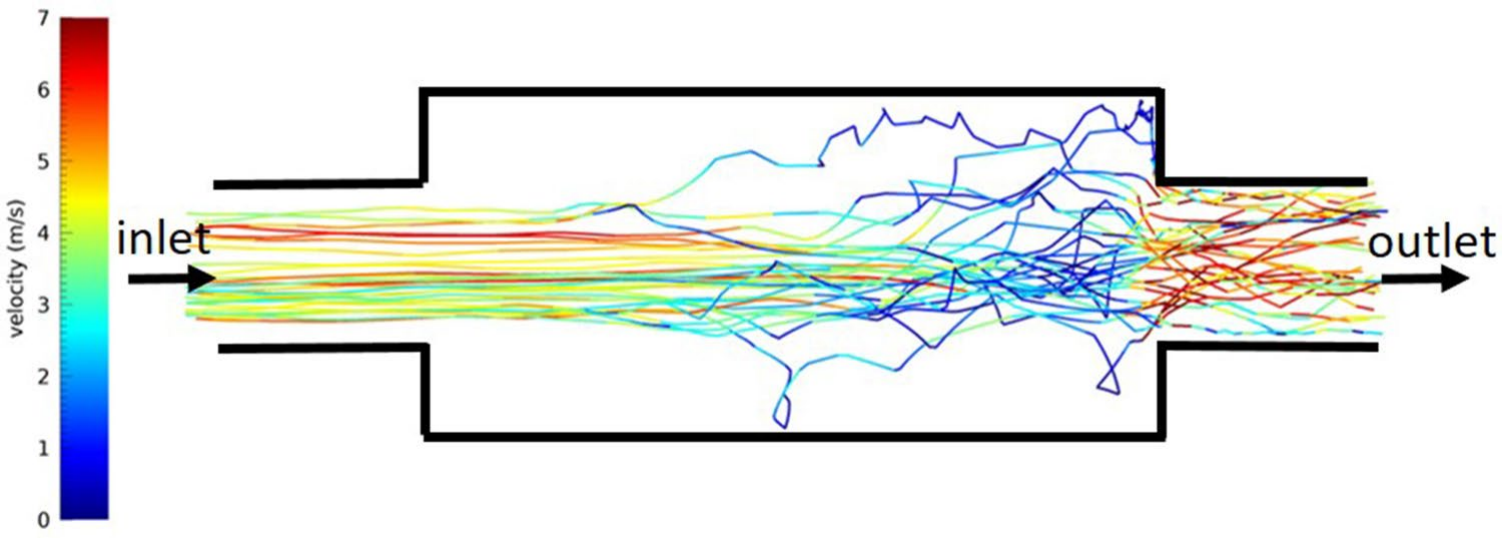
Trajectories of some particles inside the cylindrical air duct for the 230 $\mathrm{m^3}/\textrm{h}$ flux. The increased diameter produces a slowing down of the particles’ velocity in the second part of the enlarged section and a turbulent trajectory of some particles. This figure is representative of both the considered air fluxes and the particles’ sizes. The figure size is not in scale for visualisation purposes.

**Figure 6:**
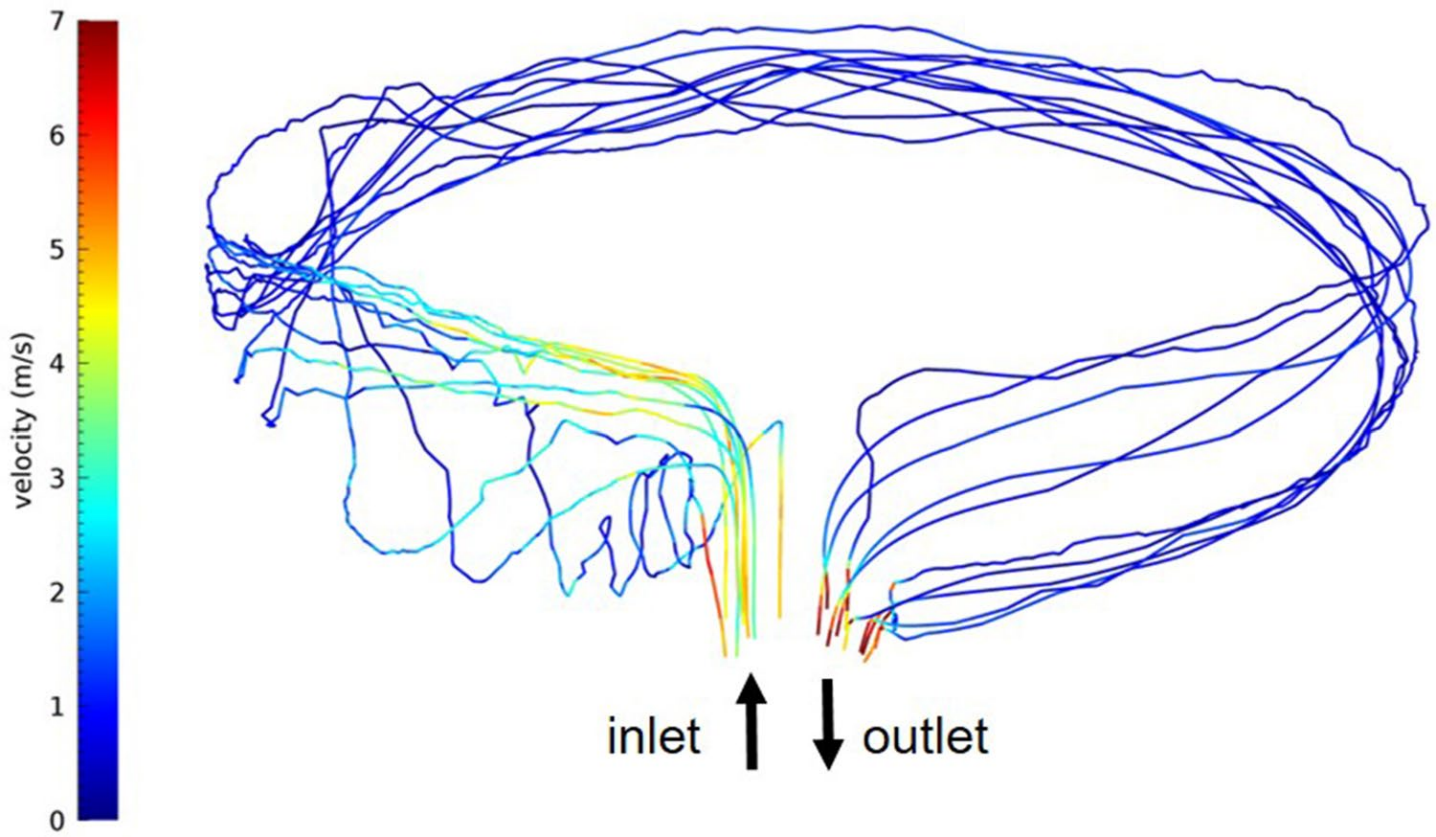
Trajectories of some particles inside the annular air duct for the 230 $\mathrm{m^3}/\textrm{h}$ flux. Particles from the smaller air duct experience some turbulent flow when entering the larger annular duct. At the considered flows, the particle trajectories return to a laminar regime. This figure represents the considered air fluxes and the particles’ sizes.

The geometries considered are a cylindrical duct in the Sun’s tracking concentrator case and an annular square-section duct in the case of the static concentrator. The volumes simulated have been discretised into fine meshes of ~1e$+$6 elements, considering the external diameters of the UVC filter of 3 m and the filter size of 28 cm (see Table 1). Figures 5 and 6 show examples of simulated droplet trajectories for the two geometry cases. The results of the CFD simulations can be considered pretty accurate within the limits of the model setup. All CFD runs have converged to the desired values of the residuals (under 1e−4/1e−6, depending on the equation). The model has been set following a preliminary analysis of the phenomenon to be modelled, hence an “a priori” determination of the Reynolds and Knudsen numbers for the flow and Reynolds and Stokes numbers for tracked droplets.

#### Optical simulations

**Figure 7:**
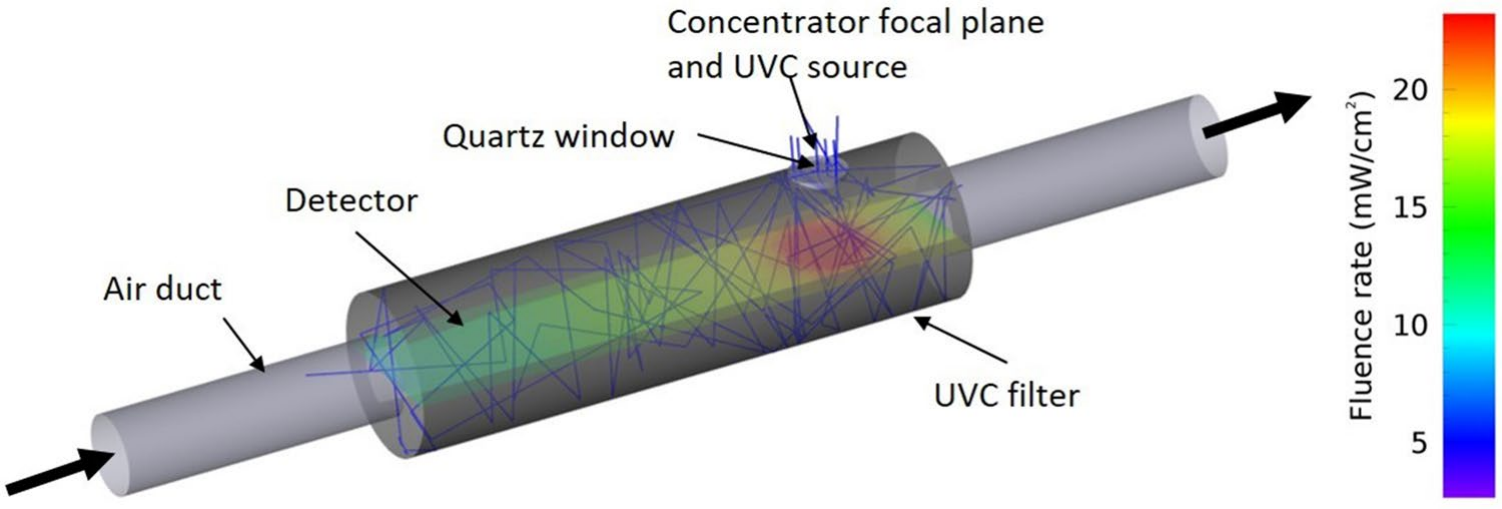
Cylindrical UVC filter. The image shows how the light rays are reflected ad scattered by the internal surface. The coloured plane is one of the volumetric detectors used to calculate the Fluence Rate inside the filter.

The optical simulations have been carried out using Zemax OpticStudio^®^. The Sun’s rays have been emitted from a source with a mean Irradiance of 0.5 $\textrm{mW}/\mathrm{cm^2}$. The optimal position for the quartz window, through which the solar UVC light enters the duct, corresponds to that part of the duct where the airspeed is lower (Fig. 5). The rays coming from the Sun have been reflected, refracted or absorbed by the optical elements until either the rays’ power fell below a given threshold ($1/10^{6}$ the initial power) or exited the optical system. The internal duct surfaces have been given a Lambertian scattering with different reflectivity to highlight the importance of a high value of *R*. The *FR* inside the filters has been evaluated by a volumetric detector, a three-dimensional array formed by cubic voxels, each of 1 $\mathrm{cm^3}$, to properly sample the *FR* spatial variations. Figure 7 shows how rays are reflected at the filter interior (blue lines) and one of the volumetric detectors along the longitudinal cross-section (100 $\times$ 28 $\times$ 1 voxel) to highlight the *FR* distribution. The detector is colour-coded to highlight the *FR* distribution along the horizontal section of the duct, the red colour indicating a higher local Fluence. Despite the Lambertian scattering, a higher *FR* is located closer to the UVC source. The fact that the maximum UVC flux is located where the airspeed is lower (Figs. 5 and 7) improves the disinfection efficiency. The simulation results, in terms of Fluence rate inside the UVC filter, can be assumed with an uncertainty below a few per cent. An adequate sampling of the fluence rate inside the filter due to the scattering distribution has been guaranteed by a sufficiently high number of starting rays from the source^105^. The optical parameters of the UVC filter components, such as the quartz Transmissivity and the PTFE Reflectivity, have been taken from datasheets, which are considered as good reference values.

#### Fluence calculation

**Figure 8:**
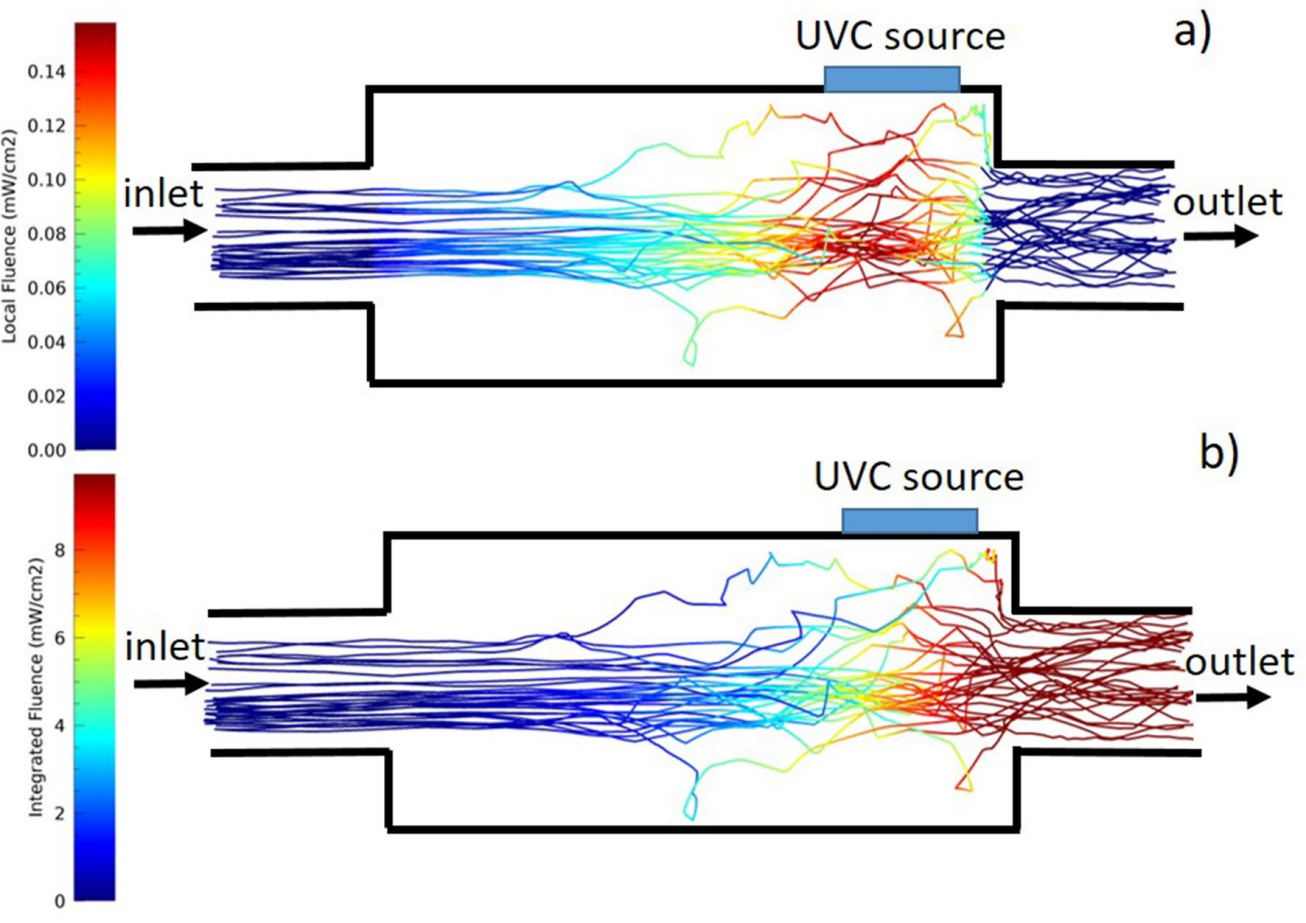
(**a**) Trajectories of the same particles of Fig. 5, inside the cylindrical air duct for the 230 $\mathrm{m^{3}}/\textrm{h}$ flux. In the second part of the filter, the reduced particles’ velocity and the higher Fluence Rate in the same region (Fig. 7) produce the local Fluence to increase. (**b**) Integrated Fluence for the same particles as the upper figure. The two figures’ sizes are not in scale for visualisation purposes.

The CFD and optical simulations have been combined to obtain the Fluence inside the UVC filter for the different parameters listed in Table 1. The following assumptions have been considered:

- each particle path inside the filter has been considered independently. The local particle’s velocity has been transformed into a residence time inside a unit volume cell (1 $\mathrm{cm^3}$);
- the residence time *t* has been multiplied by the local Fluence Rate $FR_L$, to obtain the locally delivered Fluence $F_L$ in each cell (Fig. 8a); the total delivered Fluence to the particle $F_P$ is the sum of the local Fluence along the particle’s trajectory (Figure 8b);
- the overall *F* to be used in the pathogen-dependent survival fraction calculations shown in Eq. 1 is the average value of all the particles’ $F_P$.

Figure 8a shows the locally delivered Fluence in each unit volume cell $F_L$ along the path of a little particles’ sample. It is clear that in correspondence to the UVC source, where the particle velocity is low, the local delivered Fluence is higher, while it is lower at the entrance and at the exit of the optical cavity, where the particles are faster and *FR* lower. In the same way, the total particle delivered Fluence $F_P$ quickly increases in correspondence with the low-speed region up to values of the order of the ones reported in Table 2. Figure 8b shows the total particle delivered Fluence $F_P$, pretty homogeneous for the different particles at the filter exit region, making it reasonable to consider the average value as a good estimation of the overall *F*.

## Results and discussion

Table 2 lists the expected delivered *F* for the two concentrator types and the different parameter values used in the simulations. It is evident that a high air duct internal reflectivity produces an efficiency boost for the considered solar light concentrator schemes. This should be the crucial parameter for the R &D given possible system implementation. The CFD simulations show that the pathogens’ sizes produce an almost negligible difference in the result due to the relatively high air velocity inside the filter. Thus, the main value for the different size cases has been reported.

**Table 2:** Delivered overall Fluence computed considering the two different concentrator designs (case column), internal reflectivities and air fluxes.

Case	Air flux ($\mathrm{m^3/h}$)	Air duct reflectivity R	Fluence ($\mathrm{J/m^2}$)
Tracking solar concentrator	230	0	11
		0.90	95
		0.95	162
		0.99	378
	460	0	6
		0.90	48
		0.95	81
		0.99	189
Static solar concentrator	230	0	18
		0.90	161
		0.95	210
		0.99	287
	460	0	9
		0.90	81
		0.95	105
		0.99	143

Further optimisation of the parameters depending on the system requirements can increase the device’s efficiency. However, this operation would necessitate some requirements trade-off, such as the device mass. Concerning the Sun tracking concentrator, even a relatively small size of the primary mirror (1 m), combined with a highly reflective UVC filter, could deliver a high Fluence, enough for an effective airborne pathogens’ inactivation. The concentrator requires a tracking system, which can be very simple thanks to the low Sun’s apparent speed (even a stepped tracking system could be used). However, it would still require some shrewdness to avoid contamination of lunar powder (Regolith). A bigger primary mirror or a smaller obscuration fraction (or no obscuration in the case of an off-axis telescope) would increase the delivered Fluence linearly with the increased collecting area. The mirror’s reflectivity represents another example. Even if this value is already high, a higher *R* will increase *F* by a few per cent. The air duct’s internal surface reflectivity increase would significantly contribute more. It could be helpful to boost the reflectance efficacy with proper coating by limiting the operating spectral range of the system to UV. For example, $R=0.99$ would increase the delivered *F* by a factor of 2 compared to the $R=0.95$ case. Multilayer mirrors made by a stack of HfO$_2$ and SiO$_2$ thin films have been demonstrated to reach $R=0.99$ at 250 nm^111,112^. Few materials are suitable for optimising the coating in the selected spectral range. Their deposition technology and stability in space environment over time still represent a technological challenge^113^, so a specific development project needs to be carried out. Other modifications of the UVC filter geometry, such as length, diameter and shape, would also result in increased performance through the increase of turbulence and thus the air residence time. Concerning the static concentrator, the increase of the annuls diameter or of the exit aperture (Fig. 4) is proportional to the rise of the collector size (Annulus diameter, Entry aperture, length). Proper considerations over the overall mass and size would come into play. Other possible trade-offs could concern alternative CPC designs with better collecting light efficiency^114,115^. Table 3 reports the doses for a $D_{90}$ reduction (90% or Log1) value for some airborne pathogens. The values should be compared to the expected delivered *F* from SAILOR Moon, listed in Table 2. The system would provide, for viruses, a dose sufficient for a $D_{90}$ reduction or even more. For some bacteria or fungi, which are less susceptible to UVC light exposure, some configurations would deliver a UVC dose not permitting a complete $D_{90}$ inactivation rate, which could be required for a healthy permanence of the astronauts inside the habitable modules. Anyway, it must be considered that space-based outposts will have a closed re-circulating air circuit. At every cycle, the survival fraction would be on the remnants of the previous one and would drop exponentially, cycle after cycle.

**Table 3:** Required UVC doses for a 90% reduction rate for some airborne pathogens. The values range considers the different variants of the same pathogen.

Organism	Species	Type	$D_{90}$ Range (J/m$^2$)	$D_{90}$ (J/m$^2$)
Virus				
Adenovirus		dsDNA	–	59^116^
Coronavirus		ssRNA	–	3^116^
SARS-CoV-2		ssRNA	–	5^117^
Coxsackie		ssRNA	–	21^27^
Influenza A		ssRNA	–	19^27^
Bacteria				
*Bacillus subtilis*		Veg	–	14^118^
		Sp		149^119^
*Burkholderia cepacia*		Veg	–	22^120^
*Escherichia coli*		Veg	–	11^121^
*Francisella tularensi*s		Veg	–	288^122^
*Mycobacterium* spp	*Mycobacterium tuberculosis*	Veg	5–63	5^123^, 63^123^
*Pseudomonas* spp	*Pseudomonas aeruginosa*	Veg	3–4	3^124^, 4^125^
*Serratia* spp	*Serratia marcescens*	Veg	115–209	115^124,125^
	*Serratia indica*	Veg		209^126^
*Staphylococcus* spp	*Staphylococcus aureus*	Veg	20–52	20^118^
	*Staphylococcus epidermidi*s	Veg		29^124^
	*Staphylococcus albus*	Veg		52^127^
*Streptococcus* spp	*Streptococcus pyogenes*	Veg	1–5	1^128^
	*Streptococcus agalactiae*	Veg		5^128^
Fungi				
*Candida* spp	*Candida auris*	–	–	50^117^
*Aspergillus* spp	*Aspergillus versicolor*	Sp	32–5400	32^124^
	*Aspergillus amstelodami*	Sp		870^128^
	*Aspergillus versicolor*	Veg		940^118^
	*Aspergillus niger*	Sp		5400^128^

It must be considered that this paper’s goal is to present to the scientific community an idea, still at a preliminary stage. Some simplifications assumed in the present study will be addressed in future developments. In this respect, concerning the presence of airborne microorganisms inside the lunar outposts, an important consideration regards the microgravity environment of the ISS, where transmission dynamics could happen differently than on Earth.

On the Moon, gravity is about 1/6 of the terrestrial one, and it must still be determined if the conditions will be more similar to Earth or ISS.

Moreover, in the simulations, we have considered only the UVC band for calculating the system’s delivered F. Synergic use of UVC with longer wavelength UV bands, whose irradiance is higher (Fig. 1), could help increase the pathogens’ inactivation rate even more, particularly for RNA-based viruses, as suggested in some recent works^84,129^. Experimental tests on pathogens’ inactivation efficacy using a wider bandwidth of the solar spectrum through a solar lamp are foreseen in the next future.

In modelling the dynamics of droplets, some aspects have not been taken into account. The droplets have not been considered electrically charged, and the complex dynamics of splashing/rebounding/coalescence of the droplets have been omitted. Charged droplets have been hypothesised as tending to adhere to the walls of the filter to discharge themselves, as happens, for example, with dust, which is also strongly affected by electrostatic phenomena. In this case, the droplets would be more exposed to radiation than discharged droplets. The coalescence between droplets has also been neglected, but even that would lead to the formation of larger drops, therefore more subject to the force of gravity and consequently more likely to settle. The deposition certainly involves a longer exposure to ultraviolet radiation. The drops have been considered to have elastic collisions on the walls without splashing. This hypothesis simplifies the simulations but is also conservative since a droplet that adheres to the wall at least partially after the splashing phenomenon is exposed to the radiation for a longer time, leading to a greater F received. However, despite the simulations considering a simpler estimate regarding results from a computational point of view, they are conservative in terms of performance.

It must be considered that the two components of SAILOR Moon, the concentrator and UVC filter, will be part of a more complex system which will comprehend, as a minimum, air ventilation and dust filtering. In particular, the lunar dust, called regolith, covers the lunar surface^130^, and it is composed of various types of particles of different sizes, which can be subjected to electrostatic levitation produced by the solar hard-UV and X-ray radiation. The dust will deposit over the reflecting surfaces of the concentrator, reducing the system’s efficiency. A shaking system or an electrostatic capture^131^ could help mitigate this issue. Also, the dust brought inside the habitable modules after moonwalks will be a problem for astronauts’ health^132,133^. High Efficiency Particulate Air (HEPA) filters^134^ or electrostatic facilitators^135^ could be placed before SAILOR Moon to perform dust filtering. During the next stages of this project, a Reliability, Availability, Maintainability, and Safety (RAMS) assessment will be done, and these issues will have to be addressed.

## Conclusions

We are preparing for the longer duration spaceflights necessary to enter the era of crewed planetary exploration, with the increase of “people” who are expected to participate in space missions and the rise of space missions in number and duration in the future. Recycled air, and water, purification will be the goal of future studies on the usefulness of the UVC radiation from a natural (solar) source in complex microgravity environments where the re-circulation of these media must necessarily occur for a very long time with the absolute impossibility of exchange with primary air (water).

We have presented the SAILOR Moon project, a safe, effective and sustainable solution in view of prolonged human-crewed missions on the Moon. It exploits the natural and never-ending solar UVC source for air disinfection of the future habitable modules at the lunar poles. These locations seem unique due to the slow Sun’s apparent motion and the high percentage of exposure to solar light. The project is still in the preliminary phase. The goal is to present a possible alternative to the other germicidal systems to the scientific community. The Sun tracking concentrator approach we have presented is a telescope-like limited FoV tracking concentrator with reduced optical quality and pointing accuracy requirements since the goal is to concentrate light and not produce a Sun’s image. The static concentrator requires no moving part or electric power to collect light. The simulations on its efficiency show a good performance on pathogens’ inactivation with the chosen parameters and could increase with the system optimisation. The next steps will be a feasibility study, prototyping the optical concentrators and pathogens’ inactivation performance tests on high reflective air ducts to validate the simulations. Moreover, by collecting a different wavelength band, the concentrator could also find applications other than the UVGI. For example, UVA light could stimulate some biological functions, such as favouring the growth of hydroponic cultures, vitamin D production, or simply delivering visible light for natural internal illumination with reduced use of fragile glass windows. In this case, the multilayer dielectric coatings could be used to optimise the system for high efficiency in those spectral ranges. In the cases of the absence of solar illumination, both on the Moon and for different environments such as spacecraft or Mars outposts, the concept of the highly reflective ducts could be used for air disinfection with artificial UVC sources, as it is done on Earth.

## Data Availability

The datasets generated during the current study are available from the corresponding author on reasonable request.
